# Supplementary material for: AT-101 Enhances the Antitumor Activity of Lenalidomide in Patients with Multiple Myeloma
Source: Cancers (Basel). 2023 Jan 12;15(2):477. doi: 10.3390/cancers15020477 (PMC9857228; doi:10.3390/cancers15020477)
Supplement: Supplementary file 1 [file cancers-15-00477-s001.zip › cancers-1998922-supplementary.pdf]

Supplementary Figure S1

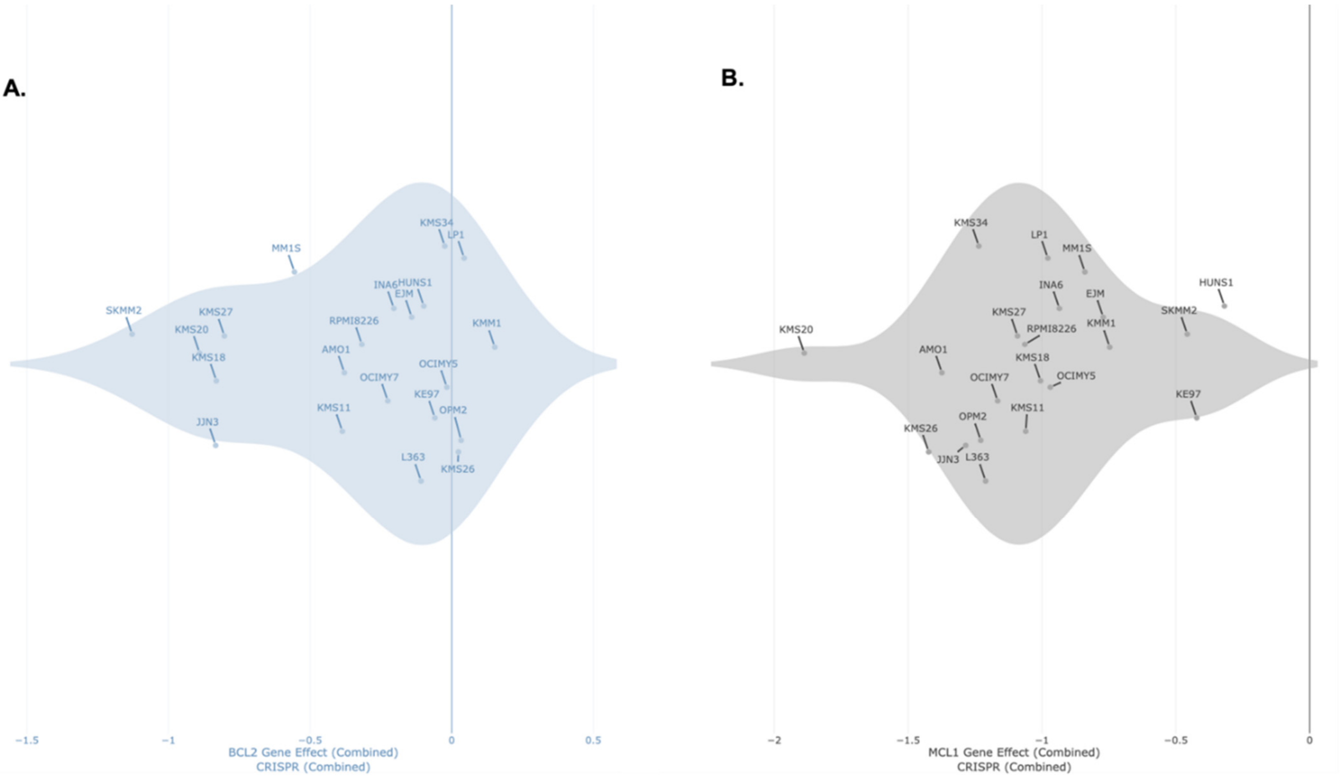

Supplemental Figure S1. BCL2 and MCL1 are essential for MM cell survival.

The CancerDep Map (Broad Novartis) was mined for determining the degree to which MM cell lines (n=29) were sensitive to CRISPR/Cas9 knockout of **A.** BCL2 or **B.** MCL1 (https://dep-map.org/portal/interactive/). A lower CERES (gene effect) score indicates higher level of survival dependency on the BCL2 or MCL1 gene knockout. A score of 0 is equivalent to a gene that is not essential whereas a score of -1 corresponds to the median of all common essential genes.

Supplementary Figure S2

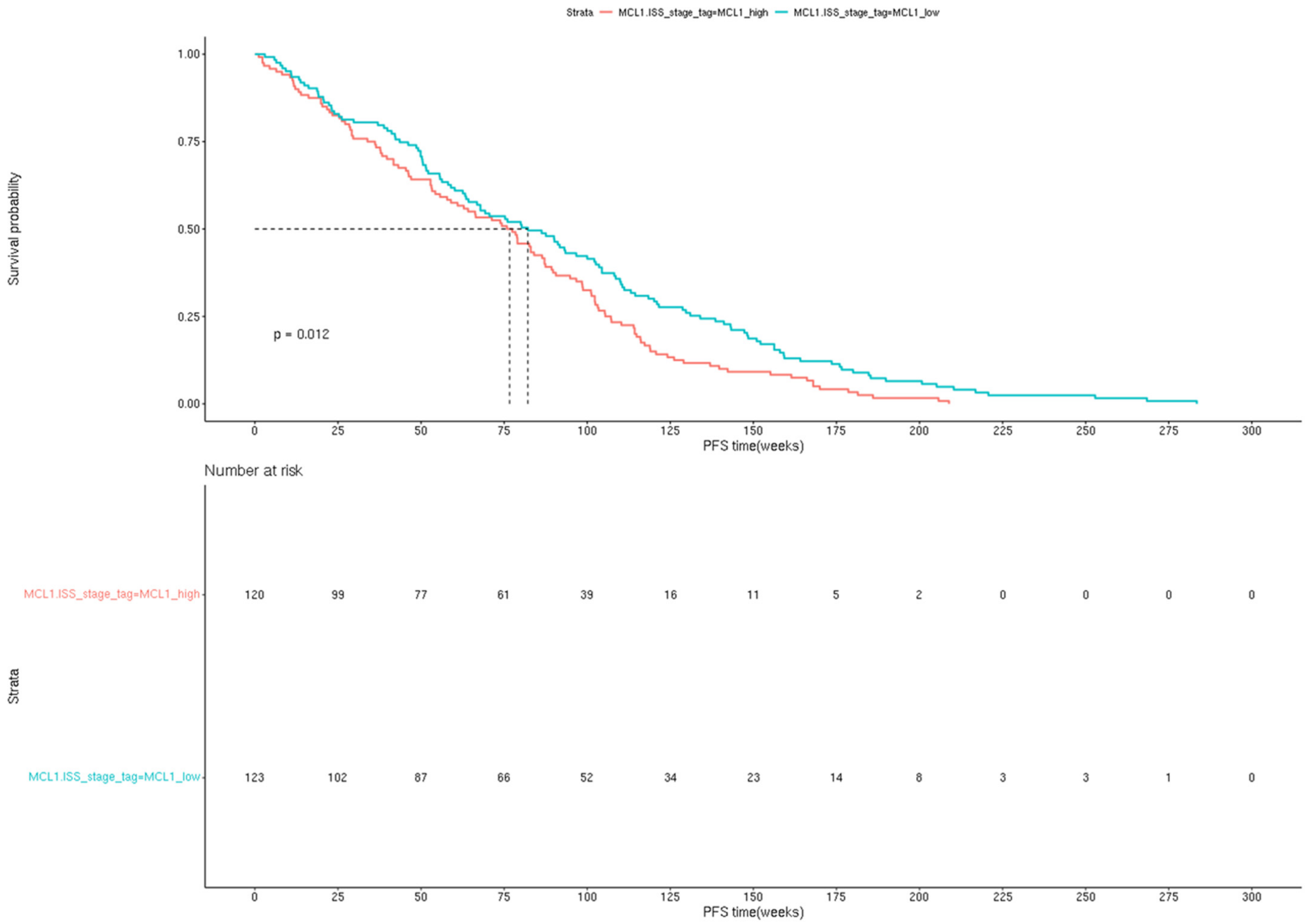

Supplemental Figure S2. MM patients with higher MCL1 gene expression have significantly shorter progression free survival.

IA13 dataset was downloaded from the MMRF CoMMpass database. Patients (n=756) with RNA expression, survival time and disease stage information were shortlisted for survival analysis. MCL1 gene expression was categorized into high/low based on the mean expression cutoff (i.e.,12.3 log2 RPKM) among patients with disease stage. Kaplan-Meier curves of MCL1 gene low/high expression groups. The risk table at the bottom indicate number of patients at risk of disease progression.

Supplementary Figure S3

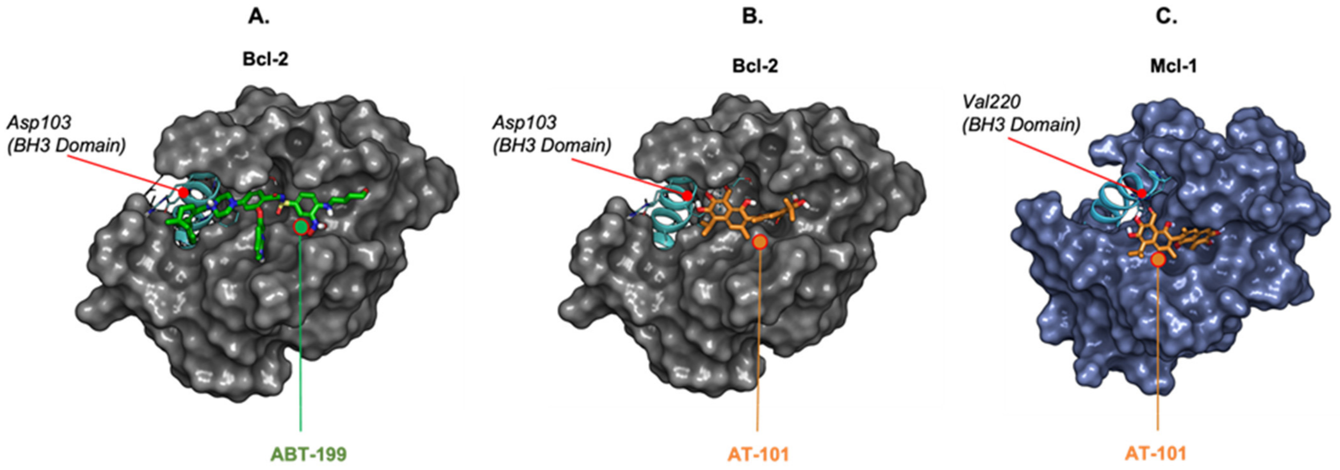

Supplemental Figure S3. AT-101 binds within the BH3 domain of Bcl2 and Mcl1.

A. Molecular dynamic simulation and reverse docking shows ABT-199 occypying the BH3 groove.

B. Comparative modeling and analysis shows that AT-101 binds to Bcl-2 and C. Mcl-1 within the BH3 groove. Its ability to bind both anti-apoptotic proteins is due to flexible binding interactions at Asp103 of Bcl-2 and Val220 of Mcl-1.

Supplementary Figure S4

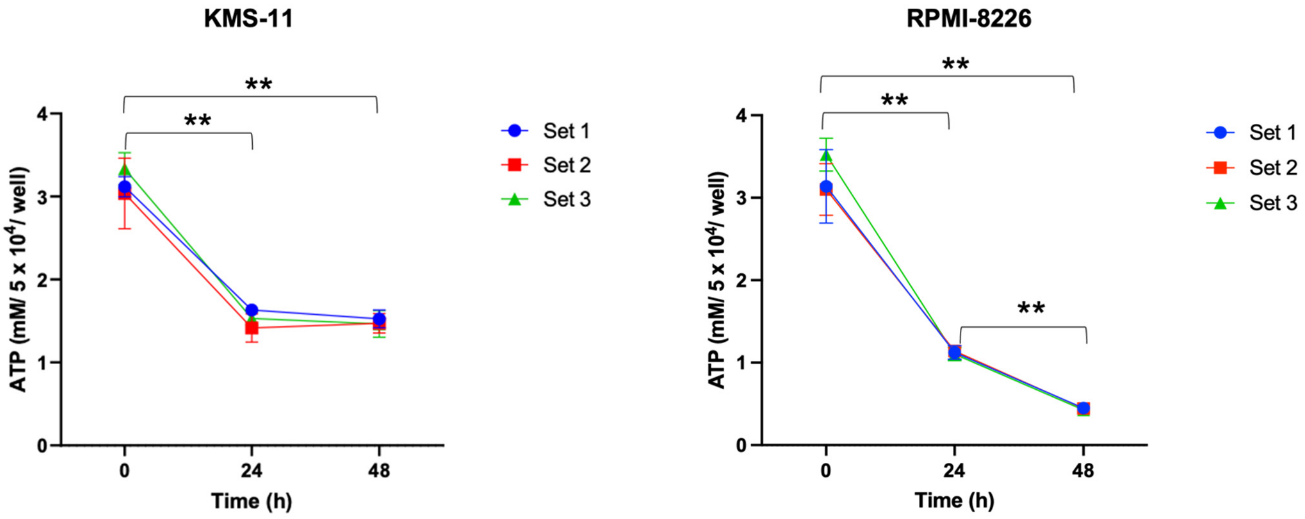

Supplementary Figure S4. AT-101 decreases ATP levels in in KMS-11 and RPMI-8226 cell lines.

Parallel to the Seahorse assay in Fig. 1G, a Luminescent ATP assay kit was used to detect the ATP concentrations in AT-101 treated KMS-11 and RPMI-8226 cells. The assay was performed 3 times (Sets 1, 2, 3) with triplicated samples in each group.

*p<0.05; **p<0.001.

Supplementary Figure S5

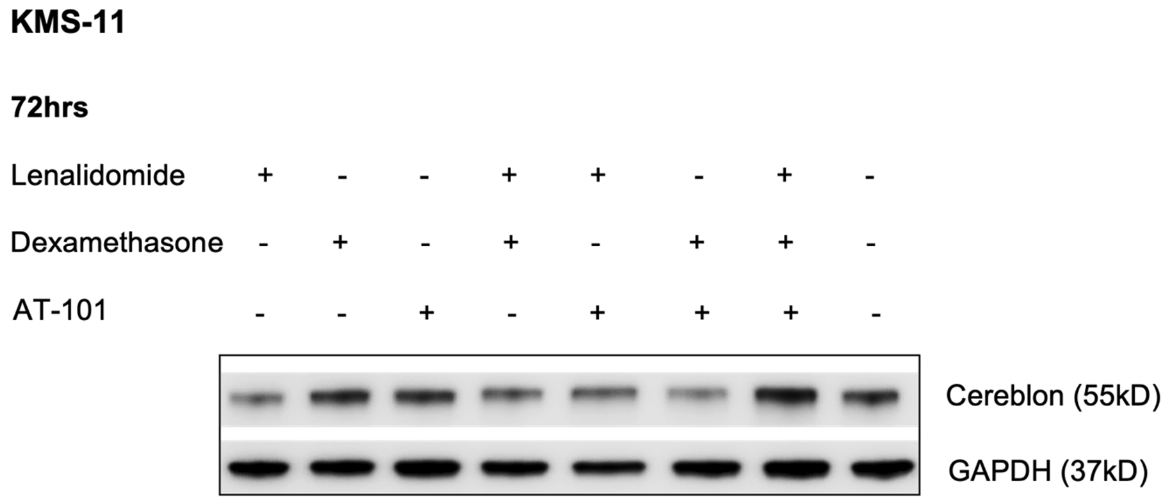

Supplemental Figure S5. Cereblon protein expression in is not affected in MM cells treated with AT-101, Lenalidomide and dexamethasone or their combination (ARd).

Western blot analysis for cereblon in KMS-11 cells treated with lenalidomide, dexamethasone, AT-101 or the combination (ARd) relative to untreated cells.

Supplementary Figure S6

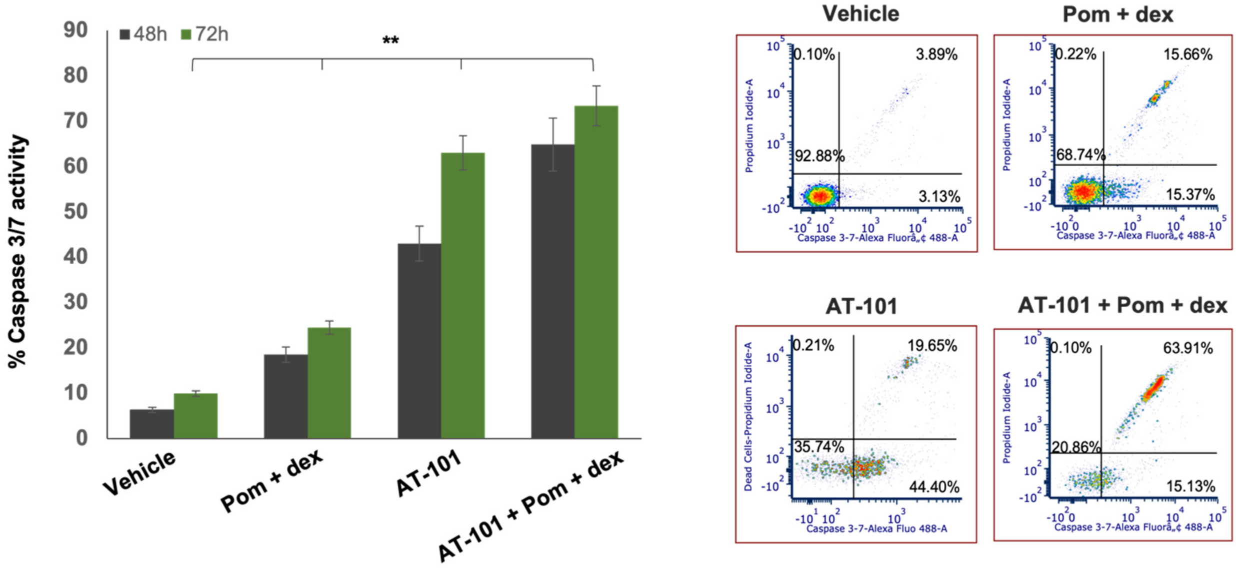

Supplemental Figure S6. Combination of Pomalidomide, dexamethasone and AT-101 activates Caspase 3/7 and induces apoptosis.

KMS-11 cells were treated with pomalidomide, dexamethasone +/- AT-101 and stained with propidium iodide and caspase3/7 activity (indicating activation of the apoptotic machinery) was measured by flow cytometry. *p<0.05; **p<0.001.

Supplemental Figure S7. Uncropped Westerm Blot Images.

Original Western Blots from Figure 3: pERK, ERK, and GAPDH

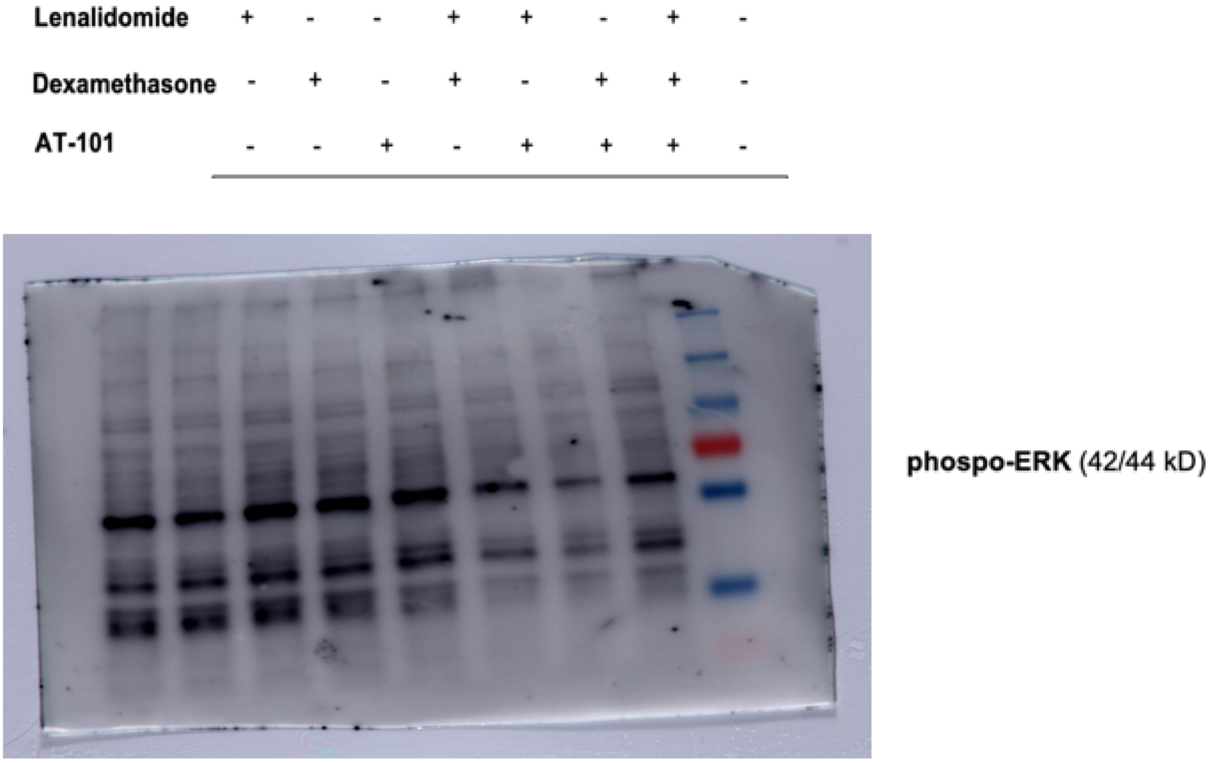

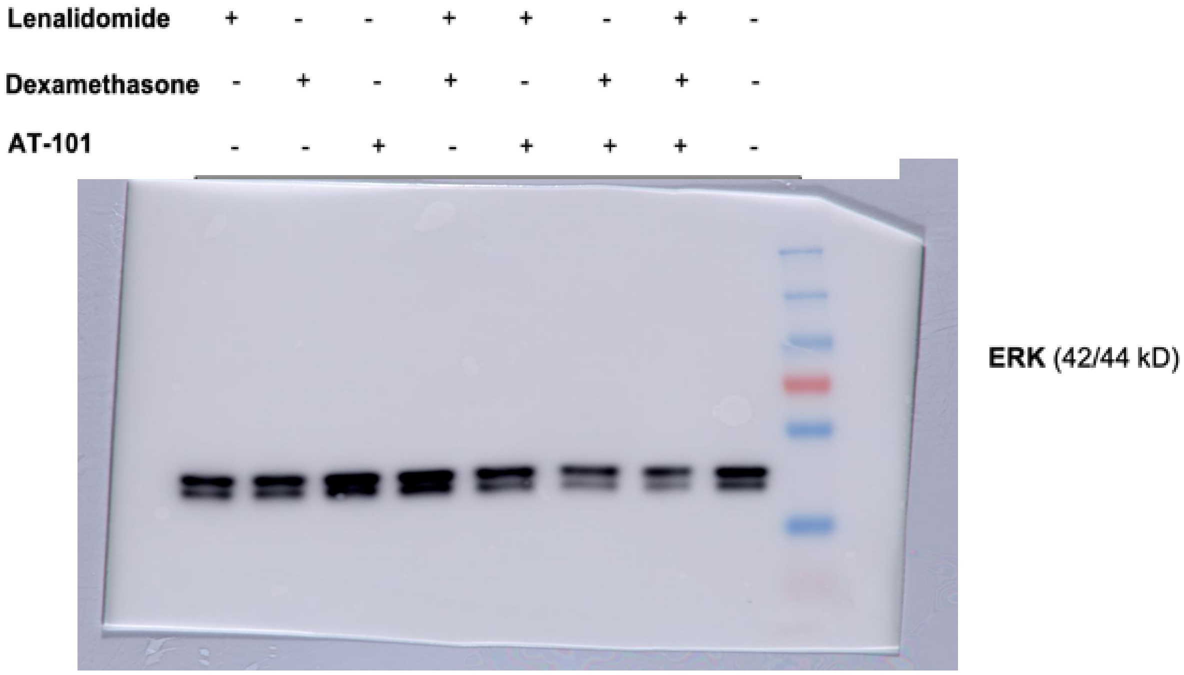

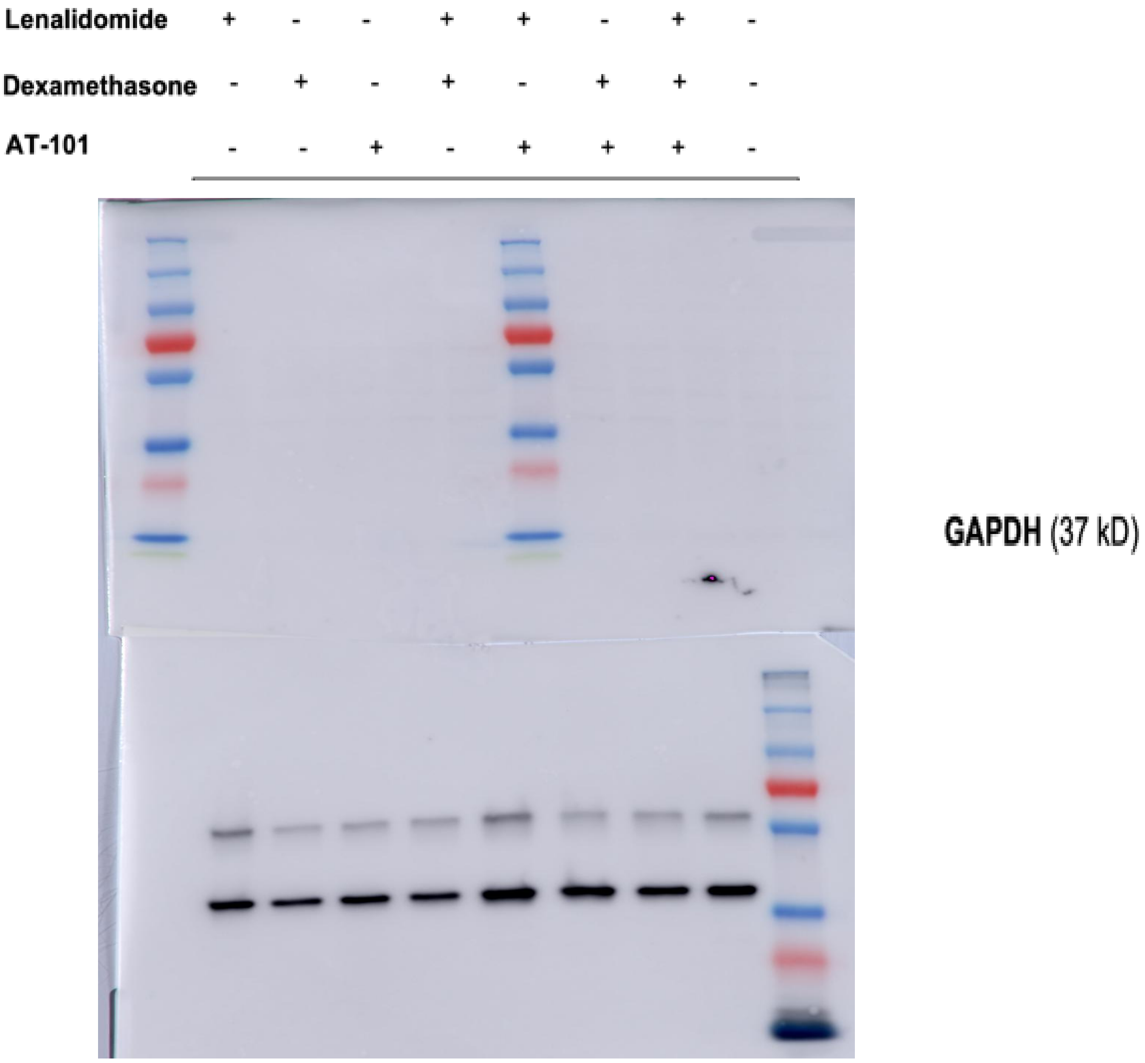
Lenalidomide + - - + + - + -
Dexamethasone - + - + - + + -
AT-101 - - + - + + + -
GAPDH (37 kD)

Original Western Blots from Supplementary

72hrs

Lenalidomide	+	-	-	+	+	-	+	-
Dexamethasone	-	+	-	+	-	+	+	-
AT-101	-	-	+	-	+	+	+	-

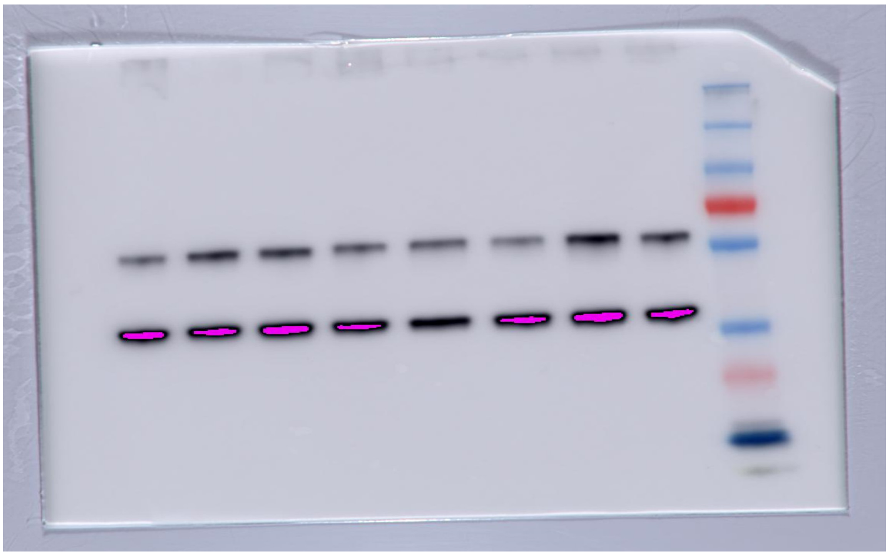

Cereblon (55kD)

72hrs

Lenalidomide	+	-	-	+	+	-	+	-
Dexamethasone	-	+	-	+	-	+	+	-
AT-101	-	-	+	-	+	+	+	-

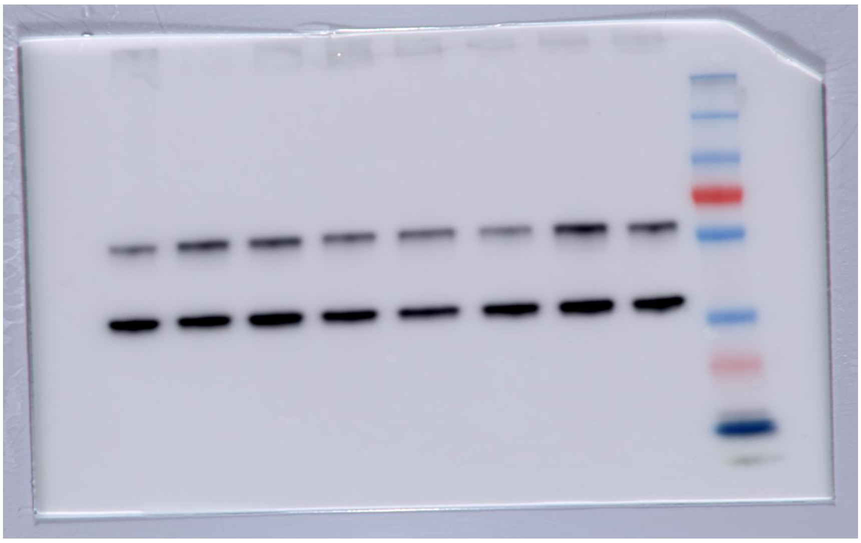

GAPDH (37kD)

Supplemental Tables.

Supplemental Table S1. RNA sequencing data evaluating expression of Bcl-2 and Mcl-1 in multiple myeloma cell lines.

Cell line primary name	A. Bcl-2 expression (RNA-seq)	B. Mcl-1expression (RNA-seq)
**AMO-1**	4.711904708	8.71945943
**EJM**	4.293136369	6.982230896
**HuNS1**	3.425718658	6.29455106
**JJN-3**	5.301124111	7.261235981
**KARPAS-620**	5.066914471	6.186873355
**KE-97**	3.415118544	5.367118893
**KHM-1B**	3.817770926	7.039892959
**KMM-1**	2.064129126	7.774808068
**KMS-11**	4.661632093	7.42740339
**KMS-12-BM**	4.680866194	5.9225247
**KMS-18**	5.583418876	7.082744347
**KMS-20**	3.900690134	8.627504829
**KMS-21BM**	4.083901768	8.078350852
**KMS-26**	5.322584919	6.903220545
**KMS-27**	4.475660586	6.992010391
**KMS-28BM**	4.720219413	6.189048742
**KMS-34**	2.06561631	6.946936429
**L-363**	4.476749294	8.363363198
**LP-1**	6.310016657	8.368890736
**MM1-S**	3.605222903	6.55070217

**MOLP-2**	5.931664145	8.462122918
**MOLP-8**		8.350282192
**NCI-H929**	1.869750743	8.683139728
**OPM-2**	3.484262625	7.865217684
**RPMI 8226**	3.248541886	5.750290926
**SK-MM-2**	7.418008276	4.924914778
**U266B1**	4.47908984	7.822288696
**Median Log2 (RPKM)**	4.47620494	7.082744347
	**Data A**	
**Data A vs. Data B: 2 tailed T test type 1**	**1.75496E-08**	
**Data A vs. Data B: 2 tailed T test type 2**	**5.87873E-12**	

Supplemental Table S2. CRISPR/Cas9 loss-of-function genetic screens in the CancerDep Map database evaluating relative essentiality of Bcl-2 and Mcl-1 towards myeloma cell survival.

DepMap ID	BCL2 Gene Effect (Combined) CRISPR (Combined)	filter	Cell Line Name	Primary Disease	Lineage
**ACH-000838**	-0.378851	multiple_myeloma	AMO1	Myeloma	Plasma Cell
**ACH-000821**	-0.140983628	multiple_myeloma	EJM	Myeloma	Plasma Cell

**ACH-000829**	-0.098667523	multiple_mye-loma	HUNS1	Myeloma	Plasma Cell
**ACH-000512**	-0.204149511	multiple_mye-loma	INA6	Myeloma	Plasma Cell
**ACH-000653**	-0.833181406	multiple_mye-loma	JJN3	Myeloma	Plasma Cell
**ACH-000167**	-0.05929296	multiple_mye-loma	KE97	Myeloma	Plasma Cell
**ACH-000889**	0.152198206	multiple_mye-loma	KMM1	Myeloma	Plasma Cell
**ACH-000714**	-0.385957453	multiple_mye-loma	KMS11	Myeloma	Plasma Cell
**ACH-000658**	-0.830808083	multiple_mye-loma	KMS18	Myeloma	Plasma Cell
**ACH-000426**	-0.89003669	multiple_mye-loma	KMS20	Myeloma	Plasma Cell
**ACH-000588**	0.023735411	multiple_mye-loma	KMS26	Myeloma	Plasma Cell
**ACH-000576**	-0.802533817	multiple_mye-loma	KMS27	Myeloma	Plasma Cell
**ACH-000541**	-0.024338286	multiple_mye-loma	KMS34	Myeloma	Plasma Cell
**ACH-000183**	-0.108108521	multiple_mye-loma	L363	Myeloma	Plasma Cell
**ACH-000204**	0.044110058	multiple_mye-loma	LP1	Myeloma	Plasma Cell
**ACH-000763**	-0.555077969	multiple_mye-loma	MM1S	Myeloma	Plasma Cell
**ACH-000854**	-0.017021595	multiple_mye-loma	OCIMY5	Myeloma	Plasma Cell

**ACH-000436**	-0.225539436	multiple_myeloma	OCIMY7	Myeloma	Plasma Cell
**ACH-000024**	0.033431894	multiple_myeloma	OPM2	Myeloma	Plasma Cell
**ACH-000817**	-0.317171749	multiple_myeloma	RPMI8226	Myeloma	Plasma Cell
**ACH-000363**	-1.128350346	multiple_myeloma	SKMM2	Myeloma	Plasma Cell
**-0.204149511**	**MEDIAN**				
**-0.3212664**	**AVERAGE**				
**DepMap ID**	**MCL1 Gene Effect (Combined) CRISPR (Combined)**	**filter**	**Cell Line Name**	**Primary Disease**	**Lineage**
**ACH-000838**	-1.373767672	multiple_myeloma	AMO1	Myeloma	Plasma Cell
**ACH-000821**	-0.77111667	multiple_myeloma	EJM	Myeloma	Plasma Cell
**ACH-000829**	-0.318604586	multiple_myeloma	HUNS1	Myeloma	Plasma Cell
**ACH-000512**	-0.934892458	multiple_myeloma	INA6	Myeloma	Plasma Cell
**ACH-000653**	-1.2849555	multiple_myeloma	JJN3	Myeloma	Plasma Cell
**ACH-000167**	-0.421815805	multiple_myeloma	KE97	Myeloma	Plasma Cell

**ACH-000889**	-0.746939035	multiple_mye-loma	KMM1	Myeloma	Plasma Cell
**ACH-000714**	-1.060659022	multiple_mye-loma	KMS11	Myeloma	Plasma Cell
**ACH-000658**	-1.005201336	multiple_mye-loma	KMS18	Myeloma	Plasma Cell
**ACH-000426**	-1.887804267	multiple_mye-loma	KMS20	Myeloma	Plasma Cell
**ACH-000588**	-1.423094781	multiple_mye-loma	KMS26	Myeloma	Plasma Cell
**ACH-000576**	-1.091293198	multiple_mye-loma	KMS27	Myeloma	Plasma Cell
**ACH-000541**	-1.235957483	multiple_mye-loma	KMS34	Myeloma	Plasma Cell
**ACH-000183**	-1.210432101	multiple_mye-loma	L363	Myeloma	Plasma Cell
**ACH-000204**	-0.97809477	multiple_mye-loma	LP1	Myeloma	Plasma Cell
**ACH-000763**	-0.83967386	multiple_mye-loma	MM1S	Myeloma	Plasma Cell
**ACH-000854**	-0.968870574	multiple_mye-loma	OCIMY5	Myeloma	Plasma Cell
**ACH-000436**	-1.165451257	multiple_mye-loma	OCIMY7	Myeloma	Plasma Cell
**ACH-000024**	-1.228588393	multiple_mye-loma	OPM2	Myeloma	Plasma Cell
**ACH-000817**	-1.064041949	multiple_mye-loma	RPMI8226	Myeloma	Plasma Cell
**ACH-000363**	-0.457387865	multiple_mye-loma	SKMM2	Myeloma	Plasma Cell

**-1.060659022**	**median**				
**-1.022316313**	**average**				

Supplemental Table S3. Genes associated with mitochondrial function that are altered in KMS-11 cells treated with AT-101.

**Bioset vs Biogroup common genes record downloaded from Correlation Engine(2021/07/09 16:08:42)**					
**https://mayo.ce.basespace.illumina.com/c/search/bg/?type=bioset&id=1649365**					
**AT-101 vs. vehicle RNA Seq. KMS11 filtered(Genes:1158) vs. REACTOME_RESPIRATORY_ELECTRON_TRANSPORT(Genes:78) Common Genes:(11)**					
**Gene**	**EntrezGene ID**	**Description**	**Imported ID**	**Rank**	**Fold Change**
**NDUFB7**	**4713**	**NADH dehydrogenase (ubiquinone) 1 beta subcomplex, 7, 18kDa**	**ENSG00000099795**	**36**	**4.1583**
**NDUFA3**	**4696**	**NADH dehydrogenase (ubiquinone) 1 alpha subcomplex, 3, 9kDa**	**ENSG00000170906**	**156**	**3.2109**
**UQCR11**	**10975**	**ubiquinol-cytochrome c reductase, complex III subunit XI**	**ENSG00000127540**	**212**	**3.0631**

**NDUFA11**	**126328**	**NADH dehydrogenase (ubiquinone) 1 alpha subcomplex, 11, 14.7kDa**	**ENSG00000174886**	**214**	**3.061**
**UQCR11**	**10975**	**ubiquinol-cytochrome c reductase, complex III subunit XI**	**ENSG00000267059**	**270**	**2.9835**
**COX8A**	**1351**	**cytochrome c oxidase subunit VIIIA (ubiquitous)**	**ENSG00000176340**	**273**	**2.9814**
**UQCRQ**	**27089**	**ubiquinol-cytochrome c reductase, complex III subunit VII, 9.5kDa**	**ENSG00000164405**	**298**	**2.9465**
**COX6A1**	**1337**	**cytochrome c oxidase subunit VIa polypeptide 1**	**ENSG00000111775**	**308**	**2.9383**
**NDUFA13**	**51079**	**NADH dehydrogenase (ubiquinone) 1 alpha subcomplex, 13**	**ENSG00000186010**	**313**	**2.9282**
**COX5B**	**1329**	**cytochrome c oxidase subunit Vb**	**ENSG00000135940**	**356**	**2.8859**

NDUFA7	4701	NADH dehydrogenase (ubiquinone) 1 alpha subcomplex, 7, 14.5kDa	ENSG00000167774	356	2.8859
NDUFA7	4701	NADH dehydrogenase (ubiquinone) 1 alpha subcomplex, 7, 14.5kDa	ENSG00000267855	379	2.86
ETFB	2109	electron-transfer-flavoprotein, beta polypeptide	ENSG00000105379	407	2.8304
Bioset vs Biogroup common genes record downloaded from Correlation Engine(2021/07/09 16:56:50)					
https://mayo.ce.basespace.illumina.com/c/search/bg/?type=bioset&id=1649582#bgcf=true&bgtf=%22Mitochondria%22&gcmax=500&gcmin=10					
AT-101 vs. ABT-199 up genes KMS11(Genes:81) vs. WONG_MITOCHONDRIA_GENE_MODULE(Genes:217) Common Genes:(3)					
Gene	EntrezGene ID	Description	Imported ID	Rank	Fold Change
NDUFA3	4696	NADH dehydrogenase (ubiquinone) 1 alpha subcomplex, 3, 9kDa	ENSG00000170906	53	1.596
COX8A	1351	cytochrome c oxidase	ENSG00000176340	67	1.542

		**subunit VIIIA (ubiquitous)**			
**UQCR11**	**10975**	**ubiquinol-cytochrome c reductase, complex III subunit XI**	**ENSG00000127540**	**73**	**1.532**

Supplemental Table S4. Clinical characteristic of patients whose samples were evaluated in correlative analyses.

Patient	Sex	Race	Trial Start Date	Date Trial Stopped	Best Documented Response	Pre-tx sample date and cell type	Post-tx sam[illegible] date and c[illegible] type
**1**	F	W	6/6/2017	9/6/2018	PR	6/6/2017	9/14/201[illegible]
						Unsorted MNC	CD138+ ce[illegible]
**3**	M	W	1/15/2018	1/8/2019	VGPR	1/3/2018	3/12/201[illegible]
						CD138+ cells	CD138+ ce[illegible]
**5**	F	W	2/1/2018	9/30/2018	SD	1/18/2018	3/12/201[illegible]
						CD138+ cells	CD138+ ce[illegible]
**7**	M	W	6/6/2017	5/7/2018	PR	6/6/2017	8/2/2017[illegible]
						Unsorted MNC	CD138+ ce[illegible]

Supplemental Materials and Methods

Cell culture and reagents

MM cell lines KMS-11, RPMI-8226 and MM1.S were used in experiments. Cells were cultured in RPMI-1640 medium containing 10% fetal bovine serum (FBS), Antibiotic-Anti-mycotic 100x (Amphotericin B Penicillin Streptomycin (Gibco, Waltham, MA, USA). CD138+ MM cells from the bone marrow mononuclear cell fraction of patients with a confirmed diagnosis of MM or in some experiments from patients on the ARd clinical trial, were isolated in a manner as previously described by us.[1] Cell viability was always maintained at >90% and was measured with a trypan blue exclusion assay and a ViCell-XR viability counter (Beckman Coulter, Brea, CA USA). RPMI medium, penicillin, streptomycin, JC-1, and FBS were purchased from Life Technologies (Waltham, MA, USA). AT-101 was purchased from Sellekhem (Houston, TX, USA). FITC Annexin V Apoptosis Detection Kit with PI (Cat # 640914), and Cell Staining Buffer (Cat # 420201) was purchased from Biolegend (San Diego, CA, USA)

Cell Viability and Drug IC_{50}

Cell viability and 72h drug IC_{50} was measured using CellTiter Glo 2.0 as previously reported [2]. Briefly RPMI-8226 and KMS-11 cells were suspended at 0.1 x 10 6 cells/mL and dispensed in 12μL aliquots into a 384 white, flat bottom plate. AT-101 and ABT-199 were serially diluted two-fold from 25μM to 195.513nm, dispensed into the cell plate, and incubated for 72 hours. At the end of

incubation, plate was equilibrated to room temperature for 30 minutes. Equal volume of room temperature CellTiter-Glo 2.0 reagent was dispensed to the plate, mixed for 2 minutes, and incubated at room temperature for 10 minutes. Luminescence was recorded on BioTek Synergy HT.

Apoptosis assay

KMS-11 and RPMI-8226 cells were treated with 5uM AT-101 for 24h and 48h. DMSO was used as a vehicle control. After 24h and 48h cells were washed with cold BioLegend's Cell Staining Buffer, centrifuged at 900g for 5min and pellet was resuspended in Annexin V Binding Buffer supplied with the kit at a concentration of 0.25-1.0 x 107 cells/mL. 100 µL of cell suspension was transferred in a 5 mL strainer tube. 5 µL of FITC Annexin V and 10 µL of Propidium Iodide Solution were added. Cells were gently vortexed and incubated for 15 min at room temperature (25°C) in the dark. After incubation cells were centrifuged at 900g for 5 min and pellet was resuspended in 400 µL of Annexin V Binding Buffer and analyzed by flow cytometry.

Western blot protein analysis

MM Immunoblotting was performed on MM cell lysates, using antibodies against cereblon, ERK and pERK as described previously [3,2].

Seahorse Extracellular Flux Assay

KMS-11 cells were treated with 5µM ABT-199, 5µM AT-101, or DMSO vehicle control in RPMI medium with 10% FBS for 24 hours in a manner previously described by us.[4] The Seahorse XF Cell Mito Stress Test Kit (Agilent 103015-100) was used to measuring cell mitochondrial function using

the Agilent Seahorse XF96 analyzer following the manufacturer's instructions (https://www.agilent.com/cs/library/usermanuals/public/XF_Cell_Mito_Stress_Test_Kit_User_Guide.pdf). The assay uses the built-in injection ports on XF sensor cartridges to add modulators of respiration into cell well during the assay to reveal the key parameters of mitochondrial function. The modulators included in this assay kit are Oligomycin, Carbonyl cyanide-4 (trifluoromethoxy) phenylhydrazone (FCCP), Rotenone, and Antimycin.

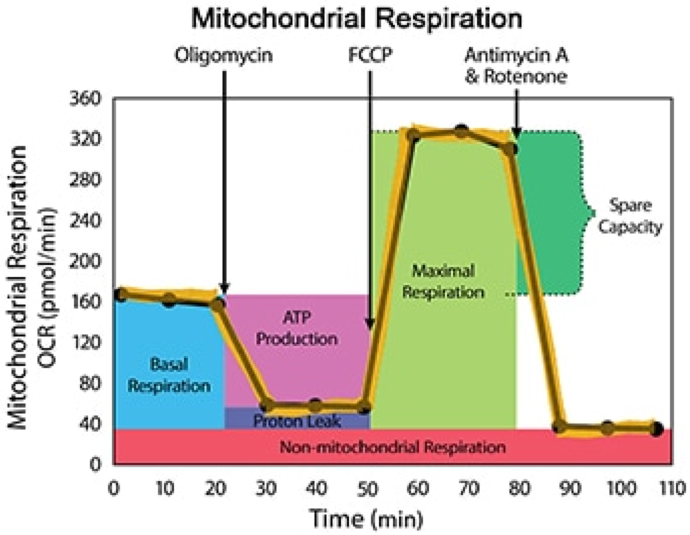

This figure illustrates the injection sequence of these modulators and the parameters can be obtained with this assay. We seeded 6x10^5 viable cells in each well and had 6 replicates for each group.

JC-1 Based Mitochondrial Membrane Potential Assay

RPMI 8226 and KMS11 cells were treated with 5μM AT-101 or vehicle control for 24 and 48 hours prior the assay. Deposited 15 μL of 2 μM CCCP or DMSO vehicle control in T-EB (300 mM Trehalose (Sigma, T0167), 80 mM KCl (Fisher Scientific, P217), 1 mM EGTA (Sigma, E-4378), 1 mM EDTA (Sigma, E5134), 0.1% BSA (thermo scientific, J30867-A1), 10 mM HEPES (Sigma H-0891) buffer pH 7.7, and 5-mM succinate (Alfa Aesar, 033386.30)) in each well of a black 384 well plate. Single cell suspensions were suspended in T-EB buffer at 4× their final density, which was 5 × 10^4 cells/well.

One volume of the 4× cell suspension was mixed with one volume of a 4× dye solution (4 μM JC-1 (ThermoFisher, M34152), 40 μg/mL oligomycin (EMD Millipore, 495455), 0.02% digitonin (Sigma, D141), 20 mM 2-mercaptoethanol (BIO-RAD, 1610710) in T-EB) to make a 2× cell/dye solution. Kept it at RT for 5 min to allow permeabilization and dye equilibration. Then added 15 μL of the 2× cell/dye mix to each treatment well, shaken for 15 s and read the fluorescence at Ex 545 nM /Em 590 nm every 5 min at RT. The $\%\Delta\Psi m = (Vmax^{AT\text{-}101}\ DMSO\text{-}Vmax^{AT\text{-}101}\ CCCP)\ x100/\ (Vmax^{Control}\ DMSO\text{-}Vmax^{Control}\ CCCP)$.

ATP assay

RPMI-8226 and KMS-11 cells were treated with 5uM AT-101 or vehicle control for 24 and 48 hours prior the assay. $5x10^5$ viable cells were seeded in each well of opaque white 96 well plate, in triplicates for each group. The assay was performed with Abcam Luminescent ATP Detection Assay Kit (ab113849) according to the manufacturers instruction and results were read on a Biotek Synergy plate reader.

RNA sequencing

RNA extraction and cDNA synthesis

The extraction of total RNA was performed in KMS-11 cells using the RNeasy Plus Mini kit (Qiagen) according to the protocol recommended by the supplier. Total RNA was quantified using a Nanodrop 1000 spectrophotometer (Thermo Scientific), quality was determined with Bio-analyzer (Agilent) followed by storage at −80 °C until needed. The reverse transcription was carried out using the Invitrogen™ SuperScript™ VILO™ Master Mix according to the manufacturer instructions.

RNA-Seq analysis

The raw RNA sequencing paired-end reads for the samples will be processed through the Mayo RNA-Seq bioinformatics pipeline, MAP-RSeq version 3.1.2 [5]. Briefly, MAP-RSeq employs the very fast, accurate and splice-aware aligner, STAR [6], to align reads to the reference human genome build hg38. The aligned reads will then be processed through a variety of modules in a parallel fashion. Gene and exon expression quantification will be performed using the Subread [7] package to obtain both raw and normalized (RPKM – Reads Per Kilobase per Million mapped reads) reads. STAR fusion algorithm [6] will be used to identify and report any expressed gene fusions in the samples. Likewise, expressed single nucleotide variants (SNVs) and small insertions-deletions (indels) will be detected using a combination of bioinformatics tools such as GATK [8], Haplotype caller [8] and RVBoost [9]. Known and novel gene isoforms will be assembled and quantified using StringTie [10] to enable detection of alternative spliced isoforms. In addition, differential exon usage will also be evaluated using DEXSeq [11] to enable comparison across conditions for alternative splicing at the exon level. Finally, comprehensive analyses will be run on the aligned reads to assess quality of the sequenced libraries. Results from all modules described above will be linked through a single html document and reported by MAP-RSeq.

Differential expression

Using the raw gene counts report from MAP-RSeq, genes that are differentially expressed between the AT-101 and ABT-199 treated cells will be assessed using the bioinformatics package edgeR 2.6.2 [12]. Genes found different between the two groups will be reported along with their magnitude of change (log2 scale) and their level of significance (False Discovery Rate, FDR < 5%). Additional

analysis was performed in Illumina Nextbio where differentially expressed genes with an FDR of <5% (alpha of 0.05) were uploaded and comparatively analyzed against datasets comprised of genes belonging to well-defined cellular pathways.

Animal studies

All animal experiments were conducted as per Mayo Clinic Institutional Animal Care and Use Committee approval and institutional guidelines. MM1.S cells (5 x 10^6) were subcutaneously injected into the flank of SCID mice. Once tumors reached a median volume of ~200mm^3, mice were randomized to receive either vehicle (PBS, intraperitoneal injection), AT-101 (35mg/kg per oral gavage), lenalidomide (10mg/kg, intraperitoneal injection) and dexamethasone (0.5mg/kg, intraperitoneal injection) or the combination of the 3 drugs (ARd), daily for 10 consecutive days. Orthotopic tumor volume was assessed by caliper measurements and quantitated by using the formula: $4/3\pi abc$; where a, b and c represent length, width and depth, respectively. Percent treatment/control (T/C %) values were calculated using the formula:

Systemic disease burden was also measure by ELISA quantifying human Ig-lambda (Hu-Igλ), which is secreted by MM1.S cells and isolated from blood sera collected from mice.

Clinical Trial

Patients

Patients with relapsed/refractory MM who required treatment as per standard guidelines [13] were considered eligible if they met the following criteria; patients had measurable disease at baseline

(including serum monoclonal protein ≥1.0 g/dL or urine monoclonal protein >200mg/24 hour or serum immunoglobulin free light chain >10mg/dL AND abnormal serum free light chain ratio), must have received 1-3 prior treatment regimens and had an absolute neutrophil count (ANC) ≥1.0 x 10^9, platelets 75 x 10^9, creatinine clearance ≥50 mL/min, and ECOG performance status ≤2. Important exclusion criteria included >3 prior treatment regimens for MM, other concurrent malignancy requiring active therapy.

Treatment of patients with AT-101

Each treatment cycle spanned 28-days with AT-101 administered once daily on days 1-21. AT-101 dosing was designed to reach a maximum daily target of 20mg (Cohort 1; 10 mg oral daily on days 1-21, Cohort 2; 20 mg oral daily on days 1-21) utilizing a standard 3 +3 dose escalation design. Notably, for cycle 1 alone, patients received single agent AT-101 on days 1-21. The rationale for this was to be able to potentially isolate both the clinical effects as well as underlying molecular mechanisms with single agent AT-101 alone. Starting with cycle 2 onwards, AT-101 was given concurrently in combination with lenalidomide 25mg oral daily on days 1-21 and dexamethasone 40mg oral on days, 1, 8 and 15. Treatment was given as outpatient for a maximum of 12 cycles. Clinical responses and disease progression were assessed by the investigator, using the International Myeloma Working Group criteria. [13] See Clinical Trial protocol for further details.

Safety assessment

Safety assessments included adverse event (AE) monitoring, vital signs, physical examination, and clinical laboratory tests. AEs were graded according to the National Cancer Institute Common Terminology Criteria for Adverse Events (CTCAE) v4.03. [14] Dose limiting toxicity was defined as an adverse event at least possibly related to treatment (definitely, probably, or possibly related) that

occurred in Cycle 2 of study treatment (first cycle of combination treatment) including grade 4 neutropenia or thrombocytopenia lasting ≥ 7 days, grade 4 infection and infestations, grade 4 hyperglycemia, other non-hematologic toxicity grade ≥ 3, and dose delay > 2 weeks.

Patients

Clinical responses as well as disease progression were assessed by the investigator, using the International Myeloma Working Group criteria. [13]

Statistical analysis

This phase I study utilized a standard cohort of three design. Patients who were eligible and received at least 1 full dose of ARd therapy were included in the analyses. Maximum tolerated dose (MTD) was defined as the dose level below the lowest dose that induces dose-limiting toxicity (DLT) in at least one-third of patients (at least 2 of a maximum of 6 patients), or the highest dose level tested if all doses are determined to be safe. The distribution of progression-free survival was estimated using the method of Kaplan and Meier. Statistical analysis was performed by SAS 9.4 biostatistical software (SAS Institute, Cary, NC). Additional details on the statistical design are provided in the Clinical Trial Protocol (Supplementary Data).

Protein complex ELISA and Immune cell analysis on primary biospecimens

CD138+ selected tumor cells or unsorted BM mononuclear cells were extracted at baseline (pre-treatment) and then after cycle 2 (post-treatment)- where one complete cycle of the ARd regimen was administered. Protein lysate was isolated from CD138+ tumor cells from MM Pts. 1, 3, 5 and 7 and Bcl-2:BIM or Mcl-1:NOXA complex formation was quantified using an advanced electrochemiluminescence assay (ECLA) system (Meso Scale Discovery [MSD]; Meso Scale Diagnostics). 96-

well Small Spot Streptavidin Sector Plates (L45SA, Mesoscale Diagnostics) were coated with 25ul/well (1ug/mL) of anti- Bcl2 (Clone 8C8, NBP233313B, Novus Biologicals) and anti-MCL1 (Clone OTI4B7, TA500984AM Origene) capture antibodies separately. 50ug of cell lysates were added into the plates and shaken at 600 rpm on BioShake IQ for 1 hour. 1:100 dilution of anti-BIM detection antibody (Clone C34C5, 2933S, Biolegend) was used for BCL2 and anti-NOXA (Clone D8L7U, 14766S, Biolegend) was used for MCL1. Plates were sealed and shaken at 600 rpm on BioShake IQ (Quantifoil Instruments GMBH) for 1 hour and washed three times with PBST (1X PBS and 0.05% Tween20) after each incubation steps. 1μg/mL of Anti Rabbit Antibody Goat SULFO-TAG antibody (R32AB-5, Mesoscale Diagnostics) was added followed by 150 uL/well of MSD Gold Read Buffer (R92TG-2, Mesoscale Diagnostics) and plates were read within 30 minutes on MESO QuickPlex SQ 120. For BM microenvironment immune cell analysis, unsorted BM mononuclear cells isolated from two of the same MM patients (Pt. 3 and Pt. 7) and one additional patient (Pt. 8) were labeled with antibodies for T-effector, T-regulatory, CD8+ cytotoxic, NK-cell and B-regulatory cells and subjected to flow-cytometry analysis as previously described by us.[15]

Patient sample preparation for Protein Complex (MSD) ELISA

Patient MNCs were sorted for CD138 (Clone DL101, 17877, Stemcell) then the cell pellets were resuspended in 1x Cell Lysis Buffer (9803, Cell Signaling Technology) containing PMSF (8553, Cell Signaling Technology).

References.

1. Paulus, A.; Manna, A.; Akhtar, S.; Paulus, S.M.; Sharma, M.; Coignet, M.V.; Jiang, L.; Roy, V.; Witzig, T.E.; Ansell, S.M.; et al. Targeting CD38 with daratumumab is lethal to Waldenstrom macroglobulinaemia cells. *Br. J. Haematol.* **2018**, *183*, 196–211. https://doi.org/10.1111/bjh.15515

2. Paulus, A.; Akhtar, S.; Caulfield, T.R.; Samuel, K.; Yousaf, H.; Bashir, Y.; Paulus, S.M.; Tran, D.; Hudec, R.; Cogen, D.; et al. Coinhibition of the deubiquitinating enzymes, USP14 and UCHL5, with VLX1570 is lethal to ibrutinib- or bortezomib-resistant Waldenstrom macroglobulinemia tumor cells. *Blood cancer journal*, **2016**, *6*, e492. https://doi.org/10.1038/bcj.2016.93
3. Paulus, A.; Chitta, K.; Akhtar, S.; Personett, D.; Miller, K.C.; Thompson, K.J.; Carr, J.; Kumar, S.; Roy, V.; Ansell, S.M.; et al. AT-101 downregulates BCL2 and MCL1 and potentiates the cytotoxic effects of lenalidomide and dexamethasone in preclinical models of multiple myeloma and Waldenström macroglobulinaemia. *Br. J. Haematol.* **2014**, *164*, 352–365. https://doi.org/10.1111/bjh.12633.
4. Paulus, A.; Akhtar, S.; Yousaf, H.; Manna, A.; Paulus, S.M.; Bashir, Y.; Caulfield, T.R.; Kuranz-Blake, M.; Chitta, K.; Wang, X.; et al. Waldenstrom macroglobulinemia cells devoid of BTKC481S or CXCR4WHIM-like mutations acquire resistance to ibrutinib through upregulation of Bcl-2 and AKT resulting in vulnerability towards venetoclax or MK2206 treatment. *Blood Cancer J.* **2017**, *7*, e565–e565, https://doi.org/10.1038/bcj.2017.40.
5. Kalari, K.R.; A Nair, A.; Bhavsar, J.D.; O'Brien, D.R.; I Davila, J.; A Bockol, M.; Nie, J.; Tang, X.; Baheti, S.; Doughty, J.B.; et al. MAP-RSeq: Mayo Analysis Pipeline for RNA sequencing. *BMC Bioinform.* **2014**, *15*, 224–224, https://doi.org/10.1186/1471-2105-15-224.
6. Dobin, A.; Davis, C.A.; Schlesinger, F.; Drenkow, J.; Zaleski, C.; Jha, S.; Batut, P.; Chaisson, M.; Gingeras, T.R. STAR: Ultrafast universal RNA-seq aligner. Bioinformatics 2013, 29, 15–21, doi:10.1093/bioinformatics/bts635.
7. Liao, Y.; Smyth, G.K.; Shi, W. The Subread aligner: fast, accurate and scalable read mapping by seed-and-vote. *Nucleic Acids Res.* **2013**, *41*, e108, https://doi.org/10.1093/nar/gkt214.
8. McKenna, A.; Hanna, M.; Banks, E.; Sivachenko, A.; Cibulskis, K.; Kernytsky, A.; Garimella, K.; Altshuler, D.; Gabriel, S.; Daly, M.; et al. The Genome Analysis Toolkit: A MapReduce framework for analyzing next-generation DNA sequencing data. *Genome Res.* **2010**, *20*, 1297–1303, doi:10.1101/gr.107524.110.
9. Wang, C.; Davila, J.I.; Baheti, S.; Bhagwate, A.V.; Wang, X.; Kocher, J.-P.A.; Slager, S.L.; Feldman, A.L.; Novak, A.J.; Cerhan, J.R.; et al. RVboost: RNA-seq variants prioritization using a boosting method. *Bioinformatics* **2014**, *30*, 3414–3416, https://doi.org/10.1093/bioinformatics/btu577.
10. Pertea, M.; Pertea, G.M.; Antonescu, C.M.; Chang, T.-C.; Mendell, J.T.; Salzberg, S.L. StringTie enables improved reconstruction of a transcriptome from RNA-seq reads. *Nat. Biotechnol.* **2015**, *33*, 290–295, https://doi.org/10.1038/nbt.3122.
11. Anders, S.; Reyes, A.; Huber, W. Detecting differential usage of exons from RNA-Seq data. *Nat. Précéd.* **2012**, https://doi.org/10.1038/npre.2012.6837.
12. Robinson, M.D.; McCarthy, D.J.; Smyth, G.K. EdgeR: A Bioconductor package for differential expression analysis of digital gene expression data. *Bioinformatics* **2010**, *26*, 139–140, https://doi.org/10.1093/bioinformatics/btp616.
13. Kumar, S.; Paiva, B.; Anderson, K.C.; Durie, B.; Landgren, O.; Moreau, P.; Munshi, N.; Lonial, S.; Bladé, J.; Mateos, M.V.; et al. International Myeloma Working Group consensus criteria for response and minimal residual disease assessment in multiple myeloma. *Lancet Oncol.* **2016**, *17*, e328-e346. https://doi.org/10.1016/s1470-2045(16)30206-6.
14. US Department of Health and Human Services Common Terminology Criteria for Adverse Events (CTCAE) v4.03. Available online: https://evs.nci.nih.gov/ftp1/CTCAE/CTCAE_4.03/CTCAE_4.03_2010-06-14_QuickReference_8.5x11.pdf (accessed on 4 April 2022).
15. Manna, A.; Kellett, T.; Aulakh, S.; Lewis-Tuffin, L.J.; Dutta, N.; Knutson, K.; Chini, E.; Pinilla-Ibarz, J.; Lamanna, N.; Manochakian, R.; et al. Targeting CD38 is lethal to Breg-like chronic lymphocytic leukemia cells and Tregs, but restores CD8+ T-cell responses. *Blood Adv.* **2020**, *4*, 2143–2157, https://doi.org/10.1182/bloodadvances.2019001091.
